# Knowledge, attitudes, and practices of health personnel in the management of diarrhea: case of the Logone and Chari Department, Far North Cameroon

**DOI:** 10.11604/pamj.2024.48.187.41974

**Published:** 2024-08-28

**Authors:** Landry Beyala, Collins Buh Nkum, Charlette Nangue, Aude Nanfak, Etienne Guenou, Ketina Hirma Tchio-Nighie, Jerome Ateudjieu

**Affiliations:** 1 Department of Health Research, M.A. SANTE (*Meilleur Accès aux Soins de Santé*), Yaoundé, Cameroon,; 2 Department of Public Health, Faculty of Medicine and Pharmaceutical Sciences, University of Dschang, Dschang, Cameroon,; 3 Division of Health Operations Research, Ministry of Public Health, Yaoundé, Cameroon

**Keywords:** Knowledge, attitudes, and practices (KAP), health personnel, diarrhea

## Abstract

**Introduction:**

management of diarrheal diseases is presented in the Integrated Management of Childhood Illnesses (IMCI) document, but is not standardized in adults. The objective of this study is to assess the knowledge, attitudes, and practices (KAP) of healthcare personnel with regard to the management of diarrhea.

**Methods:**

a descriptive cross-sectional study was conducted among health care givers in health facilities in four (4) health districts (HDs) of Logone and Chari Department of Far North Cameroon in 2016. Consenting participants, selected exhaustively in the health facilities, responded individually to a semi-structured questionnaire administered face to face on a smartphone Open Data Kit (ODK) format. Statistical analyses of the data allowed us to highlight frequencies and proportions of variables such as the definition of diarrhea, the causes of diarrhea, diagnostic techniques and methods, and the management of these diseases.

**Results:**

a total of 77 professionals participated in this study, of whom 56% were nurse assistants, 22% were nurses, and 5% were physicians; 78% of them knew the World Health Organization (WHO) definition of diarrhea. Twenty-two (29%) reported having IMCI and 82% of them practiced it. Seventy-seven (100%) reported they administered a rehydration solution according to the dehydration status of the first-line patient. Metronidazole (23%), amoxicillin (17%), and cotrimoxazole (14%) are the antimicrobials frequently prescribed in children less than 5 years of age.

**Conclusion:**

health care providers in Logone and Chari Department have different opinions regarding diarrheal diseases as well as on the management of its diseases. To improve the management of diarrhea, it would therefore be important to set up a standard document for the management of its diseases while ensuring its implementation by health professionals.

## Introduction

Diarrheal diseases remain a real public health problem, particularly serious in developing countries in general and in sub-Saharan Africa in particular; they are associated with child morbidity and mortality and are one of the most frequent reasons for consultation [1-3]. Approximately 1.7 million deaths occur each year globally due to diarrhea with the highest-burden occurring in developing countries and economically disadvantaged regions [4]. More than a quarter (26•93%) of diarrheal deaths occurred among children younger than 5 years, and about 90% (89.37%) of diarrheal deaths occurred in South Asia and sub-Saharan Africa [5,6]. In Cameroon, diarrhea represents 30% of the reasons for consultations in health facilities; the third cause of death behind respiratory infections and malaria [7]. The World Health Organization (WHO) recommends first-line management of diarrhea in children under five by continuing to feed them, increasing their fluid intake, and providing zinc supplements for 10-14 days to prevent dehydration. In addition, the WHO guidelines state that children with non-severe dehydration should “receive oral rehydration therapy (ORT) with ORS solution in a health facility”. Antimicrobials are recommended only for the treatment of bloody diarrhea or suspected cholera with severe dehydration [8,9]. In 2004, WHO and UNICEF issued a joint statement on the clinical treatment of acute diarrhea, recommending the use of low-osmolarity oral rehydration salts (ORS), zinc supplementation, increased amounts of appropriate fluids, and continued feeding [10]. The Integrated Management of Childhood Illness (IMCI) initiative was developed by the World Health Organization (WHO) with the aim of reducing childhood mortality, particularly for children under five years of age [11]. This document only provides guidelines for children under five, whereas diarrheal diseases affect both children and adults.

According to WHO, diarrhea is one of the leading causes of death in children under five years in the world. In Cameroon efforts to reduce the mortality rate of children under five due to diarrhea depend on the management of patients by medical personnel´s (nurses) in hospitals [12]. Despite the WHO guidelines of treatment for childhood diarrhea in all health facilities, very little has been achieved in rolling back the rising number of care of childhood diarrhea and related complications in one health setting. This problem could be due to lack of implementation of the prescribed treatment guidelines hence the need to assess the knowledge of nurses on the management of childhood diarrhea from the central role they play in health care delivery. Diarrhea case management can be divided into assessment, treatment, and follow-up. The assessment aims to classify patient´s level of dehydration and determine the type of diarrhea illness (acute, dysentery, or persistent diarrhea). Treatment may include oral rehydration, zinc treatment, antibiotics for selected cases, and intravenous fluids for the most severe cases. Follow-up may range from hourly observations of a severely dehydrated patient to reassessment after 4 hours for a patient with some dehydration. A patient may require follow-up after a number of days of treatment if they present with persistent or bloody diarrhea [13-15].

The Cameroon government adopted the IMCI in 1999 with the aim to reduce the mortality and morbidity of children less than five years [16]. However, diarrheal diseases affect both children and adults then how care providers can manage this disease in adults on a daily basis in health facilities? Several studies have been carried out on the community evaluation of mothers'/caregivers´ knowledge, attitudes, and practices regarding diarrhea, another evaluated knowledge, attitudes, and practices (KAP) of CHW on managing diarrhea [17,18]; in Cameroon, there are few studies on the management of cases of diarrhea by Health Care Providers (HCP) [19], even less an evaluation of its personnel on the implementation of IMCI. The literature reveals a paucity of information on the knowledge, attitudes, and practices of healthcare personnel regarding the management of diarrhea. Hence, the present study was undertaken to find out the KAPs of healthcare personnel in the Far North region of Cameroon regarding diarrhea and its management.

## Methods

**Study design:** this was a cross-sectional descriptive study carried out among health personnel of all health facilities (HFs) in four (4) health districts (HD) (Kousseri, Goulfey, Mada, and Makary) of the Department of Logone and Chari in the Far North region of Cameroon. Data on health personnel knowledge, attitudes and practices were collected using a semi-structured face-to-face questionnaire on a Smartphone by a trained interviewer.

**Setting:** this study was carried out in the Far North of Cameroon, precisely in the Department of Logone and Chari, whose capital is Kousseri. This department has the particularity of being bordered by Nigeria, Chad, and Niger; it is constituted of 4 health districts (HDs), namely Kousseri, Goulfey, Mada, and Makary. These HDs receive a large number of displaced people due to the Boko Haram phenomenon and constitute a favorable terrain for the development of diarrheal diseases, with recurrent cholera epidemics and an incidence of diarrheal diseases of 31.2% according to the *Enquête démographique et de santé et à indicateurs multiples (EDS-MICS)*, 2011 [20]. This region is also facing a shortage of health personnel because its last remains mostly in urban areas such as Yaoundé and Douala and the health system is only made up of level 4, 5, and 6 health facilities [21]. The study was conducted from April to May 2016. Participants were drawn from different health facility wards in the HDs.

**Participants:** the study targeted the health personnel of the reception, hospitalization, and laboratory services of the Health Facilities (HFs) of the HDs of Kousseri, Goulfey, Mada, and Makary. All health personnel on duty and present at the time of the interviewer's visit who voluntarily agreed to participate in the study were included.

**Data sources/measurement:** a semi-structured questionnaire adapted from previous studies [22] administered face-to-face on a smartphone in Open Data Kit (ODK) format allowed us to collect data on the WHO definition of diarrhea and its causes, diagnostic procedures, and management of diarrheal diseases by trained interviewers.

**Study size:** this was an exhaustive sampling of all healthcare personnel in the reception, hospitalization, and laboratory services on duty at the time of the interviewer's visit to all the HFs in which the 4 HDs were interviewed.

**Statistical methods:** data collected on the smartphone was directly checked and corrected before being sent to the server. Its data was analyzed on CDC/WHO Epi-Info version 7 and Microsoft Excel 2007. Descriptive statistics were used to calculate the frequencies and percentages. The variables studied were the socio-professional parameters of health workers and the knowledge, attitudes, and practices of per health workers in relation to acute diarrhea.

**Ethical consideration:** all healthcare professionals were informed of the survey and their consent was obtained before interviewing. Data that could reveal the identity of participants were coded. Data collection of these surveys was done with respect to the national and international ethical regulations for participant protection, and ethical approval was obtained from the Cameroon National Ethics Committee for Human Health Research under the clearance N°2015/08/636/CE/CNERSH/SP.

## Results

**Participants:** a total of 77 health personnel participated in the study with a 100% response rate; of whom 43 (56%) were nurse assistants, 17 (22%) were nurses, and 4 (5%) were physicians and 13 (17%) of others professionals. The most represented age group was that of 26-40-year-olds (68%) and the sex ratio male to female was 3/2. The most represented health facilities were in the sixth category (Integrated Health Centers) with 54.5% (Table 1).

**Table 1:** demographic characteristics of health personnel interviewed from health facilities of 4 health districts of the Logone and Chari Department (Cameroon) from April to May 2016 (N=77)

Socio-professional characteristics	Health personnel interviewed	
	Effective (N=77)	Percentage (%)
**Age groups**		
18 -25 years old	7	9
26 - 40 years old	52	68
More than 40 years old	18	23
**Sex**		
Male	45	58
Female	32	42
**Qualifications**		
Physicians	4	5.2
Nurses	17	22.1
Nurses assistance	43	55.8
Other^2^	13	16.9
**Type and category of health facilities**		
**Public**		
Third category	24	31.2
Fifth category	4	5.2
Sixth category	42	54.5
**Private**		
	7	9.1

2 : other professionals consisting of laboratory technicians and community health workers

### Main results

**Knowledge of interviewee´s about diarrheal diseases:** when the professionals were asked about the definition of diarrhea, 61 (79%) responded that diarrhea is “the passage of three or more loose or liquid stools in 24 hours”, while the other 15 (20%) responded that diarrhea was characterized by an increased number of daily bowel movements, with large variability observed in the frequency (Table 2). Regarding the most common causes of diarrhea, the most common responses were bacterial and parasitic infections (88.3%) and 42.8% from various other causes.

**Table 2:** knowledge of health personnel interviewed from health facilities of 4 health districts of the Logone and Chari Department (Cameroon) from April to May 2016 (N=77) per occupational category

Knowledge	Health personnel interviewed									
	Physicians		Nurses		Nurses assistance		Other^2^		Total	
			n= 17	%	n= 43	%	n= 13	%	N=77	%
Definition of diarrhea	n= 4	%								
Three or more loose or liquid stools in 24 hours	4	100	15	88.2	33	76.7	9	69	61	79
Increased number of daily bowel movements	0	0	2	11.8	9	20.9	4	31	15	20
Blooding mucus stools			0	0	1	2.4	0	0	1	1
Causes of diarrhea	0	0								
Bacterial infection	4	100	14	82.4	38	88.4	12	92.3	68	88.3
Parasitic infections	4	100	14	82.4	38	88.4	12	92.3	68	88.3
Viral infection	2	50	0	0	0	0	0	0	2	2.5
Bacterial, parasitic and viral infection	4	100	7	41.2	23	53.5	10	76.9	44	57.1
Others^2^	1	25	7	41.2	20	46.5	5	38.5	33	42.8
I don't know	0	0	1	5.8	0	0	0	0	1	1.3

2 : others: food intoxications, use of medications

**Attitudes and practices regarding the management of diarrhea:** of the 77 health personnel interviewee´s, 22 (29%) said they had a diarrhea management protocol (IMCI) and 18 (82%) implemented it, while 4 (18%) did not consult it. Table 3 presents the attitudes and practices regarding diarrhea management of the health personnel per professional category. From this table, it appears that 48% of the health personnel interviewee´s perform a stool examination before any case of diarrhea is managed and 100% systematically administer a rehydration solution to the patient.

**Table 3:** attitudes and practices of health personnel interviewed from health facilities of 4 health districts of the Logone and Chari Department (Cameroon) from April to May 2016 (N=77) per occupational category

Attitudes and practices	Health personnel interviewed									
	Physicians		Nurses		Nurses assistance	Other^2^			Total	
	n=4	%	n=17	%	n=43	%	N=13	%	N=77	%
**Do you perform a stool examination before managing a case of diarrhea?**										
Yes	02	50	09	52.9	21	48.8	05	38.5	37	48
No	02	50	08	47.1	22	51.2	08	61.5	40	52
**Do you systematically administer a rehydration solution?**										
Yes	4	100	17	100	43	100	13	100	77	100
No	0	0	0	0	0	0	0	0	0	0
**Do you prescribe antibiotics to treat all diarrheas?**										
Yes	0	0	6	35.3	23	53.5	11	84.6	40	52
No	4	100	11	64.7	20	46.5	2	15.4	37	48

2 other professionals consisting of laboratory technicians and community health workers

In terms of drug administration and management, 52% of the staff surveyed systematically prescribes antibiotics in the management of diarrheal diseases, including 53.5% of healthcare assistants and 84.6% of other health professionals. Among them, 57.5% (23/40) declare: *"I prescribe antibiotics blindly since I cannot do laboratory tests to know the cause"*. Of the 48% who do not systematically prescribe antibiotics, 56.8% (21/37) say: *"I have to do it in front of certain clinical or biological signs"*. Among the staff questioned, 87% (67/77) prescribe antibiotics in children less than 5 years of age when they present a glairo-blooded or bloody diarrhea with fever against 46.7% (36/77) when they present a liquid or soft diarrhea with fever. Metronidazole (23%), amoxicillin (17%), and cotrimoxazole (14%) are the antibiotics frequently prescribed in children less than 5 years of age, and metronidazole (17%), ciprofloxacin (15%) and ceftriaxone (12%) in adults.

## Discussion

Through this study, we were able to assess the knowledge, attitudes, and practices of health personnel faced with a case of diarrhea in the Department of Logone and Chari, Far North region of Cameroon. The results of this evaluation are of paramount importance to designing an effective health education strategy and guiding the intervention areas in improving the management of diarrheal diseases and thus reducing the number of deaths due to this disease. In this study, the opinions and practices of the professionals who work directly with patients showed great variability in the approach and management of diarrhea, including the definition and the causes of diarrhea. Variations were observed among professionals working at the same health facility and among professionals of the same category, which makes the standardization of practices more difficult. This standardization could be evident in the ongoing training of healthcare professionals which will be closely aligned with previous studies [23-25].

The definition of diarrhea was heterogeneous among the respondents, with 61 (79%) of them responding that diarrhea is “the passage of three or more loose or liquid stools in 24 hours”, while the other 15 (20%) responded that diarrhea was characterized by an increased number of daily bowel movements, with large variability observed in the frequency. This discrepancy was even more evident when the groups of professionals were compared. This difference is clearly observed in the literature, which shows great variability in the definition of diarrhea [26-34]. Regarding the opinions of the causes of diarrhea assessed in this study, the most common responses were bacterial and parasitic infections (88.3%) and 42.8% from various other causes like food intoxications and use of medications. This result is according to the literature [7,8,35,36].

In terms of the attitudes and practices of health care providers in the management of diarrheal diseases, all of them routinely administer rehydration solutions to the patient according to WHO recommendations [37] and 48% of the care providers perform a stool examination before any case of diarrhea is managed. In terms of drug management, 52% of the staff surveyed routinely prescribes antibiotics in the management of diarrheal diseases, and this varies from one professional category to another, ranging from 53.5% among nurses' aides to 84.6% among other health professionals. This prescription was motivated by the following circumstances: when mucus and bloody diarrhea (87% of cases), associated infectious pathology (48%). These results are lower than those reported by Uhlen *et al*. in 2004 [35]. This study shows that metronidazole (23%), amoxicillin (17%), and cotrimoxazole (14%) are the antibiotics frequently prescribed in children less than 5 years of age, and metronidazole (17%), ciprofloxacin (15%) and ceftriaxone (12%) in adults by health care providers in the Department of Logone and Chari. These results again raise the issue of the use of antibiotic therapy in the management of diarrhea. Although WHO and other studies recommend the use of oral rehydration solutions [8,9,15] while limiting the use of antibiotic therapy, our study highlights the limitations of these recommendations.

This study had certain limitations. The study site; prey to daily attacks by the terrorist sect Boko Haram, and some health facilities in the health districts of Makary, Goulfey, and Mada could not be visited by the investigator. To overcome these problems, data were collected by telephone call to the staff of the health facilities. Similarly, the number of professionals analyzed and the number of facilities limit the generalizability of these data.

## Conclusion

Analysis of responses to a questionnaire administered face-to-face to health professionals working in health facilities in the health districts of Kousseri, Goulfey, Mada, and Makary showed that opinions and knowledge about diarrhea were divergent, varying between professionals working in the same health facility and between professionals in the same category, making it more difficult to standardize practices. Similarly, the practices of health professionals were also divergent. In view of the crucial role played by health personnel in the health care system, the KAP of health personnel should be rationalized by organizing a training program and providing simple booklets or handouts in the local language, designed by the WHO, containing information on the management of diarrhea, as well as on common diseases. These interventions could reduce mortality due to inappropriate management of diarrhea.

### What is known about this topic

- Diarrheal diseases are associated with child morbidity and mortality and are one of the most frequent reasons for consultation;
- More than a quarter (26.93%) of diarrheal deaths occurred among children younger than 5 years, and about 90% (8937%) of diarrheal deaths occurred in South Asia and sub-Saharan Africa;
- In Cameroon, diarrhea represents 30% of the reasons for consultations in health facilities.

### What this study adds

- Only 78% of health personnel in the health districts of Kousseri, Goulfey, Mada, and Makary in Far North Cameroon, know the WHO definition of diarrhea;
- Health personnel working in health facilities in the health districts of Kousseri, Goulfey, Mada, and Makary in Far North Cameroon have divergent opinions, knowledge, and practices about the management of diarrhea, varying between professionals working in the same health facility and between professionals in the same category, making it more difficult to standardize practices;
- Metronidazole, amoxicillin, and cotrimoxazole are the antimicrobials frequently prescribed in children less than 5 years of age; in adults, metronidazole, ciprofloxacin and ceftriaxone are the most commonly prescribed antimicrobials in the health districts of Kousseri, Goulfey, Mada and Makary in Far North Cameroon.
